# Genetic drift precluded adaptation of an insect seed predator to a novel host plant in a long-term selection experiment

**DOI:** 10.1371/journal.pone.0198869

**Published:** 2018-06-12

**Authors:** Liisa Laukkanen, Aino Kalske, Anne Muola, Roosa Leimu, Pia Mutikainen

**Affiliations:** 1 Section of Ecology, Department of Biology, University of Turku, Turku, Finland; 2 Department of Plant Protection Biology, Swedish University of Agricultural Sciences, Alnarp, Sweden; 3 Department of Plant Sciences, University of Oxford, Oxford, United Kingdom; 4 Institute of Integrative Biology, ETH-Zürich, ETH-Zentrum, Zürich, Switzerland; University of Arkansas, UNITED STATES

## Abstract

Host specialization is considered a primary driver of the enormous diversity of herbivorous insects. Trade-offs in host use are hypothesized to promote this specialization, but they have mostly been studied in generalist herbivores. We conducted a multi-generation selection experiment to examine the adaptation of the specialist seed-feeding bug, *Lygaeus equestris*, to three novel host plants (*Helianthus annuus*, *Verbascum thapsus* and *Centaurea phrygia*) and to test whether trade-offs promote specialization. During the selection experiment, body size of *L*. *equestris* increased more on the novel host plant *H*. *annuus* compared to the primary host plant, *Vincetoxicum hirundinaria*, but this effect was not observed in other fitness related traits. In addition to selection, genetic drift caused variation among the experimental herbivore populations in their ability to exploit the host plants. Microsatellite data indicated that the level of within-population genetic variation decreased and population differentiation increased more in the selection line feeding on *H*. *annuus* compared to *V*. *hirundinaria*. We found a negative correlation between genetic differentiation and heterozygosity at the end of the experiment, suggesting that differentiation was significantly affected by genetic drift. We did not find fitness trade-offs between *L*. *equestris* feeding on the four hosts. Thus, trade-offs do not seem to promote specialization in *L*. *equestris*. Our results suggest that this insect herbivore is not likely to adapt to a novel host species in a time-scale of 20 generations despite sufficient genetic variation and that genetic drift disrupted the response to selection.

## Introduction

Despite the obvious advantages of polyphagy with regard to food availability, most herbivorous insects feed only on a limited set of available host plants [1,2]. Somewhat counter intuitively, empirical tests for the causes and mechanisms of this host specialization (i.e. trade-offs [3]) have been disproportionately conducted with generalist herbivores [4–8] although there are some studies examining trade-offs in specialist [9] as well as oligophagous herbivores [10–12]. This bias may have lead to an overestimation of the ability of herbivores to switch hosts and may limit our understanding of the process of host specialization.

The mechanisms that affect the rate of adaptation and specialization to host plant species may differ between generalists and specialists [13,14]. For instance, the effect of primarily defensive plant secondary metabolites on specialized herbivores may be neutral or positive, whereas the effects of these same traits on generalist herbivores are commonly detrimental [15], but see [16]. Furthermore, in specialist herbivores, host shifts can be important in initiating speciation [17,18]. Therefore, examining specialization and the ability to adapt to novel host plants in species that are more specialized allows novel insights into the process of host-plant specialization and speciation and may help us understand why specialization is common in herbivorous insects.

Here we study the adaptation of the seed-eating true bug, *Lygaeus equestris* (Lygaeniae, Heteroptera), which primarily utilizes a toxic host plant *Vincetoxicum hirundinaria* (Apocynaceae). *Lygaeus equestris* is an oligophagous seed predator, but in northern and central Europe it is strongly associated with *V*. *hirundinaria*. It therefore appears to be verging on specialization on the specialist-generalist continuum, especially in populations in the northern part of its range even though it can, and occasionally does, feed on several different plant species [19–22]. Feeding on alternative plant species may ensure survival when seeds of *V*. *hirundinaria* are scarce, even though it reduces fitness of the insect [20,23–25]. The group Lygaeinae, including *L*. *equestris* has a basal adaptation to feeding on species containing toxic cardenolides, that are common in the Apocynaceae, indicated by resistance and sequestration [22]. Some of the species in the Lygaeniae (*Arocatus longiceps* and *Arocatus melanocephalus*) have lost the ability to sequester cardenolides after switching to less toxic host plants presumably either through genetic drift or negative selection on the trait, suggesting that host switching has resulted in trade-offs on their ability to feed on their ancestral hosts [22]. Similar changes in the ability to feed on the ancestral primary toxic host plant may be observed also within species after allowing populations to adapt to alternative hosts.

We conducted a multi-generation quasi-natural selection experiment (i.e. mass selection) with replicated populations of *L*. *equestris* to assess host specialization and trade-offs. We did not determine which individuals were allowed to contribute to the next generation, but the differences in contribution result from inherent differences in adaptedness to the environment [3]. We used two alternative host species that *L*. *equestris* occasionally feeds on in our study area, *Centaurea phrygia* and *Verbascum thapsus*, as well as a benign, non-toxic host, the sunflower *Helianthus annuus*, commonly used to rear this species in laboratory conditions [20,24]. In a previous cross-feeding experiment, we did not find fitness trade-offs between herbivores feeding on different host plants [25], suggesting that such trade-offs were not strong enough to affect adaptation and specialization of this herbivore to its current primary host. However, we expected to have greater power in detecting trade-offs in the current multi-generation selection experiment compared to the previous split-brood experiment conducted in only one generation [3].

In this study, we first investigated whether *L*. *equestris* adapts to novel host plant species over the course of c. 20 generations by comparing the changes in fitness that occurred during the experiment between the selection lines for primary and novel hosts. Selection generally results in loss of additive genetic variation within a population [5,26]. Therefore, we examined whether selection impacted genetic variation in our experimental replicate populations using neutral markers, and quantitative traits. We also determined whether genetic drift contributed to genetic differentiation among the replicate populations by comparing pairwise genetic differentiation between source and end populations to estimates of within population neutral genetic variation. Finally, we examined whether potential adaptation to a novel host was associated with a cost in performance when subsequently feeding on the primary host or on other alternative hosts.

## Materials and methods

### Study species

*Lygaeus equestris* L. (Heteroptera: Lygaeidae) is an oligophagous true bug specialized to feed on seeds of the long-lived perennial herb *Vincetoxicum hirundinaria* Med. (= *Cynanchum vincetoxicum* (L.) Pers.) (Apocynaceae). Both nymphs and adults feed on the green ovulae, and developing, matured, as well as already dispersed seeds [19,27]. Occasionally *L*. *equestris* also feeds on other plant parts, such as stems and leaves, of *V*. *hirundinaria* [19]. In Finland, *L*. *equestris* occurs only in association with *V*. *hirundinaria* populations, where it can be locally common [28–31]. *Vincetoxicum hirundinaria* contains several secondary compounds, such as alkaloids and phenolic compounds [32,33] that may be essential feeding stimulants of these specialized herbivores. In our study area *L*. *equestris* is usually univoltine and overwinters as adults. The females oviposit on ground layer vegetation in June and July. Adults of the new generation commonly appear from late July onwards [19].

Both observations from natural populations and laboratory experiments confirm that *L*. *equestris* occasionally feeds on several other plant species in addition to *V*. *hirundinaria* [19–21,23,25]. Especially in spring, when the seeds of *V*. *hirundinaria* from the previous year may be difficult to find, and during and after severe summer droughts, alternative host plants might be essential for the survival of *L*. *equestris* [19]. Moreover, because the nymphs are wingless and earthbound, they depend on local food supplies [19]. In this study, we used *Helianthus annuus* L. (Asteraceae), *Centaurea phrygia* L. (Asteraceae), and *Verbascum thapsus* L. (Scrophulariaceae) as alternative host-plant species. *Lygaeus equestris* is known to feed and complete its development on the seeds of these species [25,34], although they differ in terms of nutritional quality–*C*. *phrygia* and *V*. *thapsus* are relatively poor foods compared to *H*. *annuus* [25] (see Results). Both *V*. *hirundinaria* and *V*. *thapsus* are common in the study area whereas *C*. *phrygia* is relatively rare [35]. *Helianthus annuus* does not naturally occur in Finland, and its cultivation is modest, but in cultivated *H*. *annuus* fields in Central and Southern Europe, *L*. *equestris* is a common visitor and a pest [34]. Even though *H*. *annuus* is not necessarily a novel host for species of *L*. *equestris*, it is for the bugs occurring in Finland and therefore can be considered a novel host in this experiment. We had also used the seeds of *H*. *annuus* and *V*. *thapsus* as food for *L*. *equestris* in feeding experiments before and knew that the bugs can complete development on them [25].

### Establishment of selection lines

**Fig 1:**
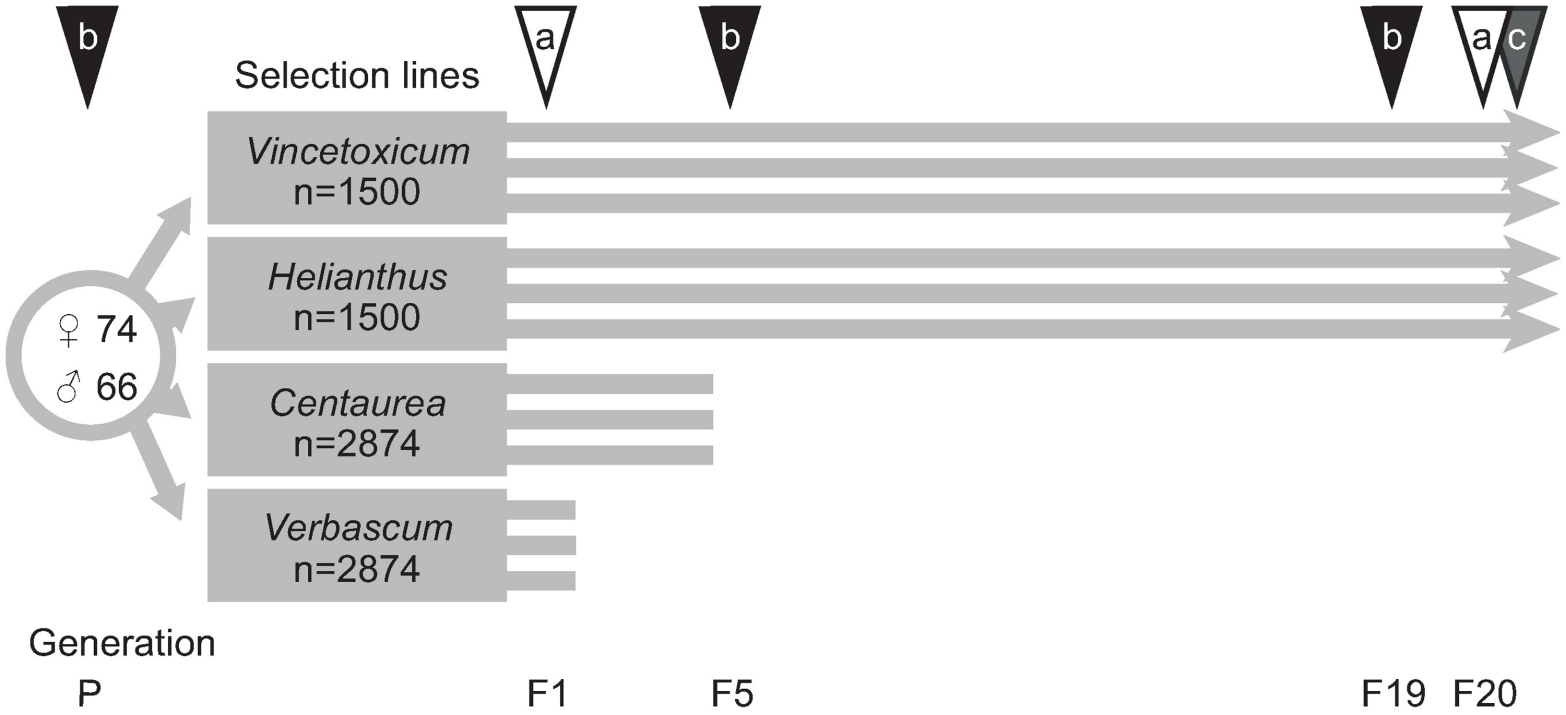
Experimental set up for the multi-generation selection experiment. We established four selection lines (*Vincetoxicum*, *Helianthus*, *Centaurea*, and *Verbascum*), each with three replicate populations as indicated by the parallel lines. The *Vincetoxicum* and *Helianthus* selection lines persisted for 26 months, whereas *Centaurea* and *Verbascum* selection lines went extinct soon after the beginning. The triangles and letters above the parallel lines indicate time points at which we performed the various feeding assays and sampled populations for genetic analysis: we a) measured the number of fertile eggs, mortality, developmental time, and adult biomass in the *Vincetoxicum* and *Helianthus* selection lines, b) sampled individuals for population genetic analysis and c) measured mortality, developmental time, and adult biomass in a split-brood experiment with insects from the two selection lines reared on all four host plant species to test for quantitative genetic variation in adaptive traits and trade-offs.

To study the adaptation of *L*. *equestris* to novel host plants, we established four selection lines where the bugs were reared either on their primary host plant (*Vincetoxicum* selection line) or on one of the three novel host plants (*Helianthus*, *Centaurea*, and *Verbascum* selection lines). We established three replicate populations in each of the four selection lines (Fig 1). We collected the parental generation of *L*. *equestris* (74 females and 66 males) from a natural population (i.e. source population) located in the SW archipelago of Finland (N 60° 14.0’, E 21° 56.8’) in August 2008. Insects were collected mostly off of *V*. *hirundinaria*, which is abundant at this site and from the ground surrounding the plants. All necessary permits for the field collections of insects were obtained from the Forest Administration of Finland (Metsähallitus). No endangered or protected species were collected. The bugs were fed with a mixture of seeds from the four host plants and allowed to breed randomly. We collected all egg clusters and randomly divided the emerging F_1_ nymphs into four groups to form the four selection lines on the first day after hatching. The groups were then further subdivided into the three replicate populations of each selection line with 500 individuals per population in the *Vincetoxicum* and *Helianthus* selection lines and 958 individuals per population in the *Centaurea* and *Verbascum* selection lines. We used more individuals in the latter two selection lines to compensate for the expected greater mortality on these host plants. The selection experiment and all the accompanying bioassays described below were performed at 30°C and a 22:2 LD photoperiod [36]. For more details on the establishment of the selection lines and replicate populations see the S1 Appendix.

We maintained the *Vincetoxicum* and *Helianthus* selection lines in the laboratory for 26 months, which equals c. 20 generations (Fig 1). The *Verbascum* selection line went extinct after the first generation because the individuals that developed on *V*. *thapsus* were sterile. The *Centaurea* selection line remained viable for longer but eventually all replicate populations went extinct after 6–9 months (3–4 generations) because fecundity in the replicate populations was very low. The adults delayed mating after completing development and we observed very few nymphs in these replicate populations. Adaptation to novel host plant was therefore only assessed for the *Helianthus* selection line. We assessed population sizes and sex ratios after 22 months of selection in *Vincetoxicum* and *Helianthus* replicate populations by counting all individuals and determining the sex of the adults. We calculated effective population sizes following the formula for non-overlapping generations [37]. The effective population sizes ranged between 227–432 and 62–307 for *Vincetoxicum* and *Helianthus* selection lines respectively (S1 Table).

We studied selection, adaptation and trade-offs using three approaches described in detail below. First, we compared the performance of *L*. *equestris* on the two successful host species in the beginning and end of the experiment (section *Adaptation to novel host plant*). We then examined changes in neutral genetic variation and population differentiation over the course of the selection experiment (section *Changes in genetic variation and population differentiation during the selection experiment*). Finally, we compared the performance of *L*. *equestris* from the two surviving selection lines on all four host plant species after selection (section *Genetic variation after selection and costs of adaptation*).

### Adaptation to a novel host plant

To investigate whether *L*. *equestris* adapted to a novel host plant (*H*. *annuus*) during the selection experiment, we examined the fitness of *L*. *equestris* on *V*. *hirundinaria* and *H*. *annuus* before and after selection. In this feeding assay, we always fed the nymphs with the seeds of the host plant of their respective selection line. Increased fitness on the host plant after the selection may indicate either herbivore adaptation to the host plant, adaptation to laboratory conditions, or both [38]. However, changes in fitness between the two selection lines would indicate adaptation of *L*. *equestris* specifically to that species on which the increase in fitness was greater.

#### Fitness before selection

We first determined the reproductive success of *L*. *equestris* feeding on the two host plants before selection. We randomly selected fifth-instar nymphs of the F_1_-generation from each replicate population of the *Vincetoxicum* and *Helianthus* selection lines. After the nymphs reached maturity, we paired them within the replicate populations. Each pair was kept in a transparent plastic container (volume 1 L) and fed with seeds of the host plant of their respective selection lines. If the male died, it was replaced with another male from the same replicate population, but if the female died we considered the pair to have finished breeding. We counted the number of fertile eggs from two to five females per replicate population, resulting in 10 and 7 females from *Vincetoxicum* and *Helianthus* selection lines respectively.

We conducted a feeding assay to monitor nymph growth from hatching to maturity on the two host plants. We used 34 egg clusters laid by the P-generation to obtain the nymphs. One nymph from each of the 34 egg clusters was assigned to feed on the seeds of *H*. *annuus* on the first day after hatching. We randomly chose 12 egg clusters out of the 34 egg clusters, and assigned one nymph of each of these 12 egg clusters to feed on the seeds of *V*. *hirundinaria*. We reared the nymphs individually in Petri dishes (diameter 9 cm) with food and distilled water *ad libitum*. We examined the Petri dishes daily to record mortality, developmental time and adult biomass on the day of the last moult.

#### Fitness after selection

At the end of the selection experiment we repeated the feeding assay for the measures of fitness of *L*. *equestris* from the two selection lines on their respective host plants. To estimate female reproductive success and to obtain offspring for the other feeding assay (see below *Genetic variation after selection and costs of adaptation*), we randomly selected fifth-instar *L*. *equestris* nymphs of the last, c. 20^th^ generation from each replicate population and placed them into individual Petri dishes. We paired the resulting adults randomly within the replicate populations and counted the number of fertile eggs of 35 and 52 females from the *Vincetoxicum* and *Helianthus* selection lines respectively. We also repeated the feeding assay with the nymphs obtained from these pairings to measure mortality, developmental time, and adult mass. The nymphs of each *L*. *equestris* family were at least half-sibs. We obtained nymphs from 2–8 females from each replicate population resulting in 22 families from the *Vincetoxicum* selection line and 12 families from the *Helianthus* selection line. In total, there were 337 individuals from 34 families.

#### Statistical analyses

To test for the adaptation of *L*. *equestris* to the novel host plant over the course of the selection experiment, we compared changes in performance from the first to last generation between the two selection lines when reared on the host plant of their respective selection lines. We included generation (1 vs. c. 20), selection line (*Vincetoxicum* or *Helianthus*), and their interaction as fixed factors in all analyses. The factor generation tests for response to selection and the interaction between generation and selection line for adaptation to the novel host plant. We conducted separate analyses for each fitness measure, i.e. number of fertile eggs, mortality, developmental time, and adult biomass. We constructed generalized linear models with negative binomial error structure and log link function for the number of fertile eggs and binary error structure for mortality (dead/alive). We analysed developmental time and adult biomass using general linear models with normal error structure and identity link function. We used family means for these two traits in the 20^th^ generation to avoid pseudo-replication. Based on Grubb’s test for outliers [39], we excluded one observation from the analysis of developmental time. We used a priori contrasts for testing pairwise differences in the herbivore fitness before and after selection within each selection line, and between the two selection lines.

### Changes in genetic variation and population differentiation during the selection experiment

We estimated genetic variation using microsatellite markers at three different time points over the course of the selection experiment. We sampled 30 adult *L*. *equestris* individuals from the source population corresponding to the P-generation in early May 2009. Since *L*. *equestris* overwinters as adults, this was the same generation that we used to establish the selection experiment in the previous fall. During the selection experiment, we sampled 30 adult *L*. *equestris* from each of the *Vincetoxicum* and *Helianthus* replicate populations at two different time points: at 7 and 25 months or c. 5 and 19 generations, respectively. We stored all samples in 94% ethanol at 4°C until DNA extraction.

#### Microsatellite discovery and genotyping

We developed 12 new microsatellite loci for *L*. *equestris* using a next-generation sequencing approach where potential microsatellite loci were first located *in silico* from 454 pyrosequencing reads, after which primers were designed and potential loci tested for PCR amplification and polymorphism. Genomic sequence data was produced by GenoScreen (France) and all testing and subsequent microsatellite genotyping was performed by the Center of Evolutionary Applications (University of Turku, Finland). For more details on the microsatellite discovery and genotyping see S1 Appendix and for microsatellite characteristics, see S2 Table.

#### Statistical analyses

We estimated allelic richness (*A*_*r*_), expected (*H*_e_) and observed (*H*_o_) heterozygosity per locus, for each replicate population and generation 0, c. 5 and c. 19 using FSTAT 2.9.3.2 [40]. We compared estimates of genetic variation between selection lines and replicate populations at the three different time points to determine how marker-based neutral genetic variation changed in the two selection lines during the selection experiment. We conducted separate repeated measures analyses for each measure of genetic variation (*A*_*r*_, *H*_e_, *H*_o_). We included selection line, replicate population nested within selection line, generation, and their interactions as fixed factors in all three analyses.

We analysed overall genetic differentiation among populations at two different time points, generation c. 5 and c. 19, separately for both selection lines using *F*_ST_ as estimated by θ [41] in R (version 3.4.1; [42]). We calculated 95% confidence intervals for these four overall *F*_ST_ estimates, and separately for the two selection lines and two time points with 1000 bootstraps (*diffCalc* in package diveRsity; [43]). To test for the effects of genetic drift on population differentiation, we estimated correlations between genetic differentiation in the source population (generation 0) and populations at generation c. 19 with their observed heterozygosity *H*_o_ at the same time point. Similar methods in studies of isolated island populations have been used to infer the effects of genetic drift on population differentiation [44,45].

### Genetic variation after selection and costs of adaptation

At the end of the c. 20 generations of selection in the *Vincetoxicum* and *Helianthus* selection lines, we conducted a split-brood feeding assay where we reared nymphs from the same family on all four host plants used in the original four selection lines and measured their fitness. This design allowed us to study 1) quantitative genetic variation in adaptive traits after the selection and 2) the potential costs of adapting to a novel host plant. Moreover, including *V*. *thapsus* and *C*. *phrygia* in comparisons of fitness allowed us to estimate whether adaptation to one novel host plant species, *H*. *annuus*, had resulted in cross-adaptation to other novel host plants [46,47].

We used *L*. *equestris* offspring from the same females as in the feeding assay assessing adaptation to host plants (see above *Adaptation to the novel host plant*: *Fitness after selection*). We assigned the nymphs randomly to feed on seeds of one of the four host plant species on the first day after hatching. We obtained on average 38 nymphs from 3–8 families per population to be assigned randomly to feed on seeds of the four host-plant species resulting in a total of 1351 individuals with 1–17 siblings per *L*. *equestris* family per food-plant species. We recorded mortality and measured developmental time, and adult biomass to estimate fitness.

#### Statistical analysis

We used three separate generalized linear mixed models to test for genetic variation and costs of adaptation with mortality, developmental time, and adult biomass as response variables. Selection line, replicate population nested within the selection line, food plant (the plant nymph fed on during the feeding assay), sex, and all possible interactions were included as fixed factors. We included *L*. *equestris* family nested within the replicate population and the interaction between family and food plant as random factors in the same analyses to examine genetic variation in adaptive traits. Sex and its interactions were included in the models for developmental time and adult biomass, but not for mortality, because we were not able to determine the sex of the juveniles. We were not able to test for the family by sex interaction, because in some of the families there were not enough replicates for both sexes. We analyzed mortality with a binomial error structure (logit link function) and developmental time and adult biomass with the normal error structure (identity link function). Because only a very low number of individuals in certain combinations of family and food plant treatment survived to adulthood, the models for developmental time and adult biomass were not estimable. Therefore, we excluded six families with less than four surviving individuals in each food plant treatment from these analyses. All these families were from different replicate populations from the *Helianthus* selection line. Similarly, as only one family from the replicate population three from *Helianthus* selection line had more than three individuals per each food plant treatment, the entire replicate population was removed from all analyses. This reduced the number of families from the *Helianthus* selection line to seven. One observation was excluded from the analysis of the developmental time based on the results of outlier test [39]. In total, we had data on mortality from 1261 individuals, developmental time from 940 individuals, and adult biomass from 942 individuals. All analyses were conducted using the GLIMMIX in SAS (version SAS 9.2; SAS Institute Inc. 2002–2007).

To further study the costs of adaptation to a single host plant, we estimated negative genetic correlations, i.e., trade-offs between fitness of *L*. *equestris* across the different food plant species after selection. We calculated Spearman rank correlations based on family means for mortality, developmental time, and adult biomass separately within the *Vincetoxicum* and *Helianthus* selection lines. For the *Vincetoxicum* selection line, we calculated all correlations between fitness values among all four host plants (54 tests altogether). For the *Helianthus* selection line, we calculated correlations between mortality on all four host plants. For developmental time and adult biomass we assessd only those between *V*. *hirundinaria* and *H*. *annuus*, as the number of surviving individuals per family feeding on *C*. *phrygia* and *V*. *thapsus* was very low (22 tests altogether). All correlation analyses were conducted with SAS (version SAS 9.2; SAS Institute Inc. 2002–2007). Bonferroni correction was used to correct for multiple comparisons separately for each of the two selection lines.

## Results

### Adaptation to novel host plant

**Fig 2:**
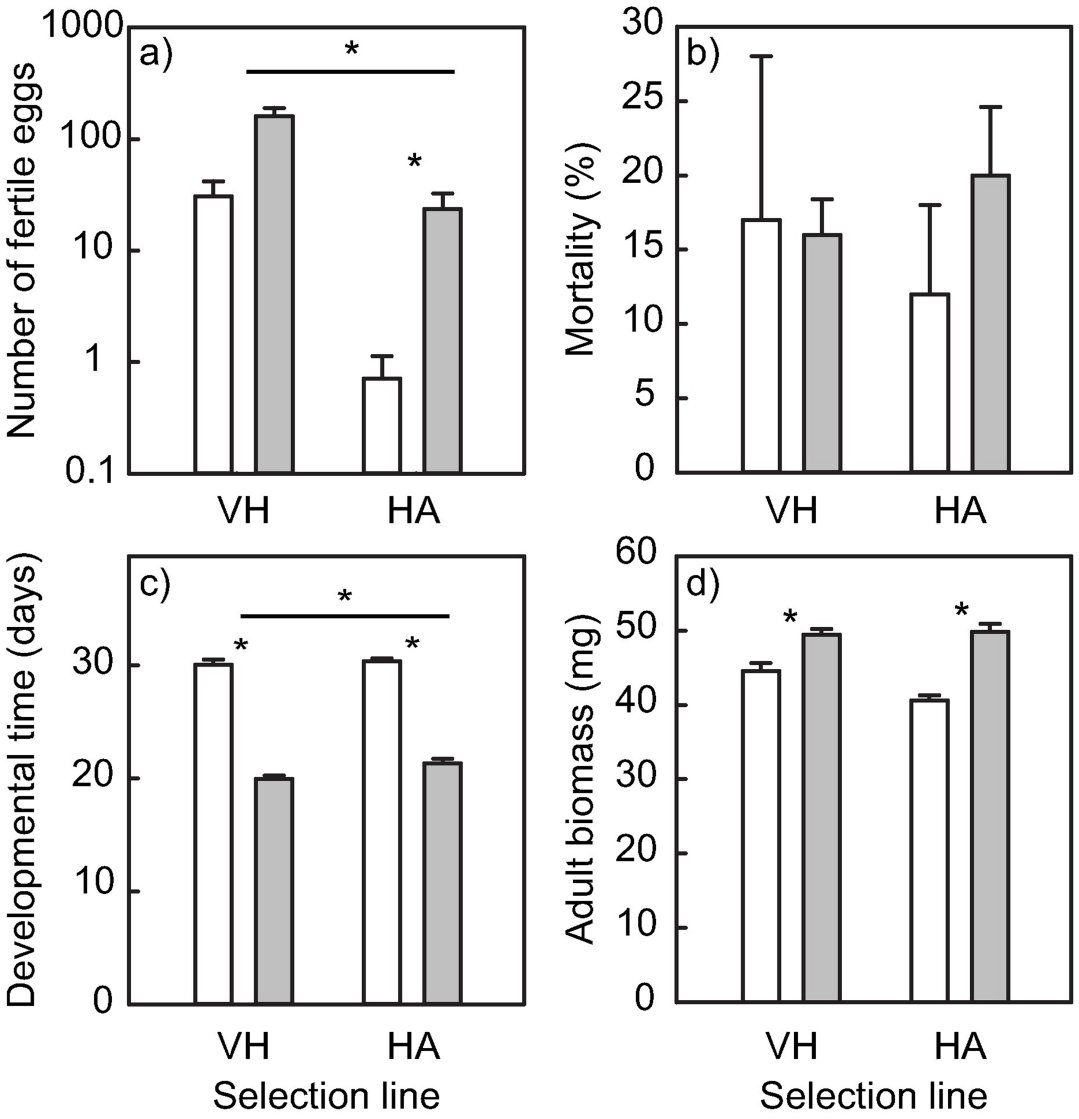
Fitness of *L*. *equestris* on the host plant of each selection line before and after selection. Fitness was measured as a) number of fertile eggs, b) mortality, c) developmental time, and d) adult biomass of *Lygaeus equestris* before (white bars) and after (grey bars) long-term selection for primary host plant *Vincetoxicum hirundinaria* (VH) or novel host plant *Helianthus annuus* (HA; least square means ± SE). Statistical significance levels were obtained using contrasts. Asterisks directly above bars indicate differences in the herbivore fitness before and after selection within each selection line. Asterisks and a line indicate differences between the two selection lines. * *P* < 0.05.

In both selection lines, the number of fertile eggs per female increased during the selection experiment (Table 1 and Fig 2a). In the *Vincetoxicum* selection line, the number of fertile eggs produced increased fourfold (contrast: *F*_1,100_ = 3.05, *P* = 0.084) whereas in the *Helianthus* selection line, the number of fertile eggs increased 32-fold over the course of the same number of generations (Fig 2a). Although reproductive success appeared to have increased more in the *Helianthus* selection line, the interaction between generation and selection line was not statistically significant (Table 1). Females from the *Helianthus* selection line produced 98% fewer eggs than those from the *Vincetoxicum* selection line at the beginning of the experiment. After selection, the difference between the selection lines was still considerable at 85% (Fig 2a). Overall, *L*. *equestris* females from the *Vincetoxicum* selection line produced more than six times more eggs compared to females from the *Helianthus* selection line (Table 1 and Fig 2a).

**Table 1:** Results of ANOVA testing for adaptation of *Lygaeus equestris* to a novel host plant in four fitness measures.

	No. of fertile eggs		Mortality		Developmental time		Adult biomass	
	Df	*F*	df	*F*	df	*F*	df	*F*
Generation	1, 100	11.90***	1, 379	0.78	1, 68	736.07***	1, 68	58.23***
Selection line	1, 100	14.39***	1, 379	0.02	1, 68	5.44*	1, 68	3.66
Selection line × generation	1, 100	1.52	1, 379	0.96	1, 68	2.38	1, 68	5.26*

Notes: The factor “Generation” tests for response to selection

* 0.01 < *P* < 0.05;

*** *P* < 0.001.

Mortality of *L*. *equestris* did not differ significantly between the selection lines (Table 1 and Fig 2b) and did not change significantly in either of the selection lines during the selection experiment (*F*_1,379_ < 0.01, *P* = 0.954, and *F*_1,379_ = 2.49, *P* = 0.115 for *Vincetoxicum* and *Helianthus* selection lines, respectively; Fig 2b). Developmental time decreased in both selection lines (*F*_1,68_ = 382.7, *P* < 0.001, and *F*_1,68_ = 353.6, *P* < 0.001 for the *Vincetoxicum* and *Helianthus* selection lines, respectively; Table 1, Fig 2c).

Adult biomass increased during the experiment in both selection lines (Table 1 and Fig 2d; *Helianthus F*_1,68_ = 53.19, *P* < 0.001, and *Vincetoxicum F*_1,68_ = 13.26, *P* < 0.001). The increase was greater (22.6%) in the *Helianthus* selection line compared to the 11.1% increase in the *Vincetoxicum* selection line indicated by the statistically significant interaction term (Table 1).

### Changes in genetic variation and population differentiation during the selection experiment

**Fig 3:**
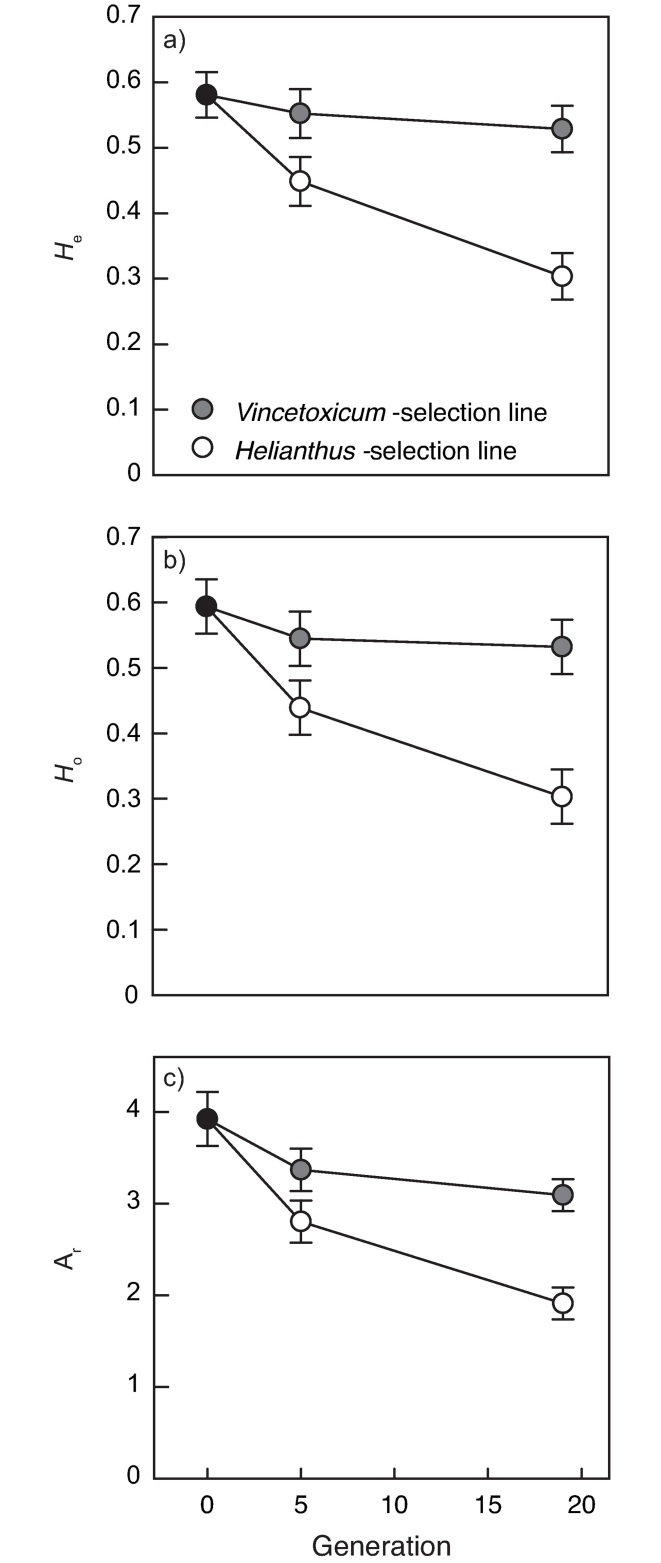
Changes in genetic variation during the selection experiment in *Vincetoxicum* and *Helianthus* selection lines. Panels are a) expected heterozygosity *H*_e_, b) observed heterozygosity *H*_o_ and c) allelic richness *A*_*r*_. Genetic variation was measured with 14 microsatellite markers. Values are least square means (± SE).

**Fig 4:**
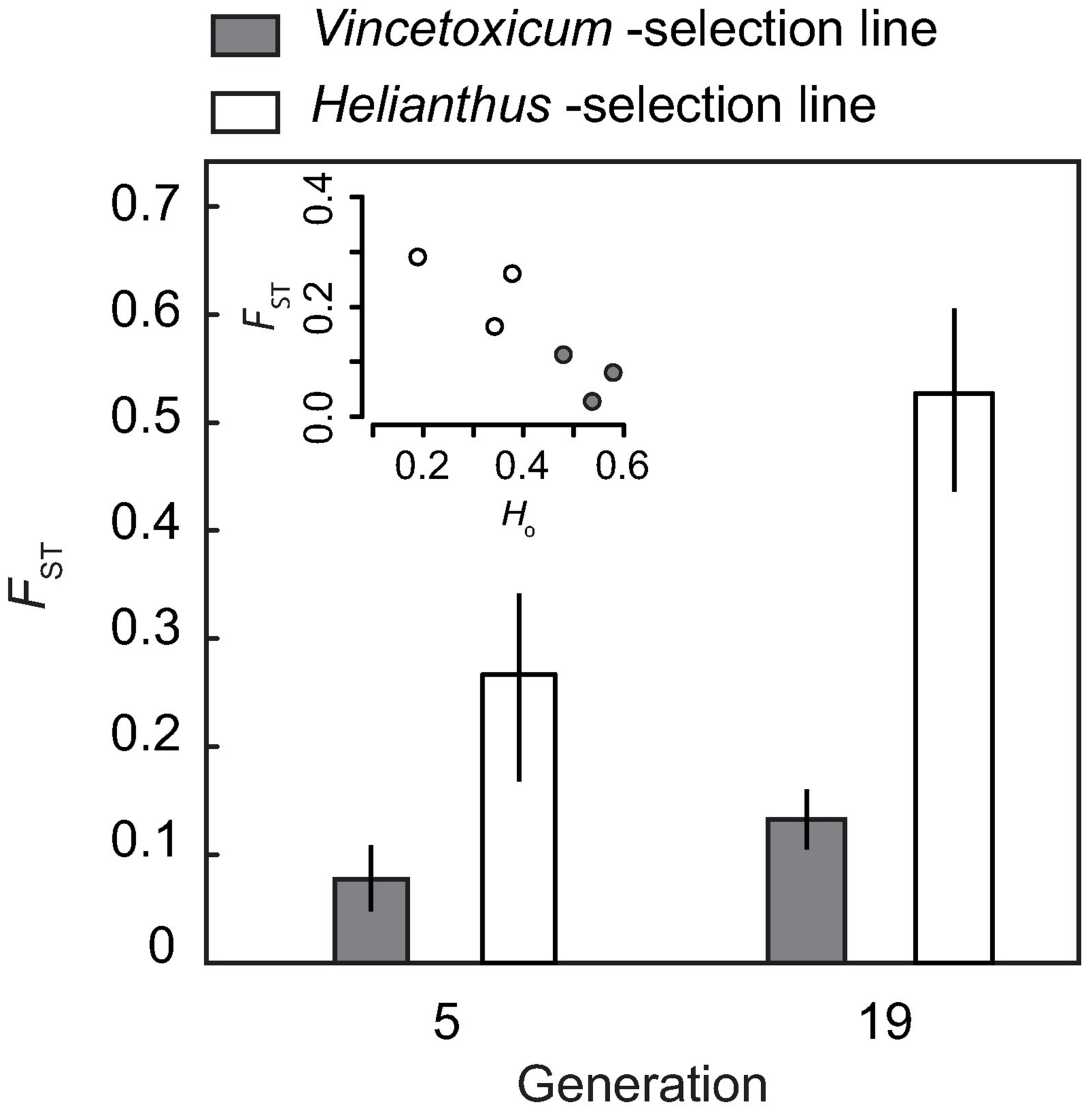
Changes in population differentiation during the selection experiment. Bars are overall *F*_ST_ values (as estimated by [41]) for the three replicate populations in each of *Vincetoxicum* and *Helianthus* selection lines at two time points with 95% confidence intervals. Sub-panel in top left corner shows the negative association between pairwise *F*_ST_ between source population (generation 0) and each population at generation c. 19 and observed heterozygosity *H*_o_.

The amount of genetic variation measured as allelic richness, and expected and observed heterozygosity, decreased during the selection experiment in the *Helianthus* selection line (Table 2 and Fig 3). The reductions in allelic richness, *H*_e_ and *H*_o_ were 30–38% greater in the *Helianthus* selection line compared to the *Vincetoxicum* selection line (Fig 3). Populations in the *Helianthus* selection lines were overall more than three times more differentiated than populations in the *Vincetoxicum* selection lines at each time point (Fig 4). Differentiation among *Vincetoxicum* populations was low at generation 5 (*F*_ST_ = 0.078) and increased by 73% to moderate at generation 19 (*F*_ST_ = 0.134; Fig 4). Differentiation among *Helianthus* populations increased by 99% from that at generation 5 (*F*_ST_ = 0.265) to generation 19 (*F*_ST_ = 0.526, Fig 4). Pairwise *F*_ST_ between the source population and generation 19 populations and population *H*_o_ were negatively correlated (r = -0.886, *P* = 0.019, Fig 4) indicating that genetic drift was an important driver of population differentiation [44,45].

**Table 2:** Results of ANOVAs testing for the effects of the selection on expected (*H*_e_) and observed heterozygosity (*H*_o_) and allelic richness (*A*_r_) in the two selection lines and the replicate populations.

	*H*_e_			*H*_o_			*A*_r_		
	df	F	P	df	F	P	df	F	P
Selection line	1, 72	5.44	0.023	1, 72	4.39	0.040	1, 72	3.44	0.068
Replicate population (sel. line)	4, 72	0.24	0.917	4, 72	0.22	0.926	4, 72	0.06	0.992
Generation	2, 71	37.92	<0.001	2, 144	34.18	<0.001	2, 71	55.01	<0.001
Selection line × generation	2, 71	17.94	<0.001	2, 144	14.33	<0.001	2, 71	9.90	<0.001
Replicate population (sel. line) × generation	8, 98.5	1.63	0.1261	8, 144	1.72	0.099	8, 98.5	0.97	0.464

### Genetic variation after selection and cost of adaptation

The mortality of *L*. *equestris* varied significantly among the four food plants indicating variation in the quality of these plant species as hosts (*F*_3,72_ = 30.75, *P* < 0.001; S1 Fig). This was also evident from the rapid extinction of the *Verbascum* selection line (see *Establishment of selection lines)*. Nearly 50% of nymphs fed on *V*. *thapsus* died during development, whereas the nymphs grown on *V*. *hirundinaria*, *H*. *annuus*, and *C*. *phrygia* were more likely to reach maturity. Mortality did not differ significantly among the replicate populations (*F*_3,1145_ = 2.03, *P* = 0.109) or selection lines (*F*_1,1145_ = 1.24, *P* = 0.265). The effect of food plants on mortality was similar for both selection lines (selection line × food plant: *F*_3,1145_ = 1.09, *P* = 0.351) and replicate populations (replicate population [selection line] × food plant: *F*_9,1145_ = 1.66, *P* = 0.094).

**Fig 5:**
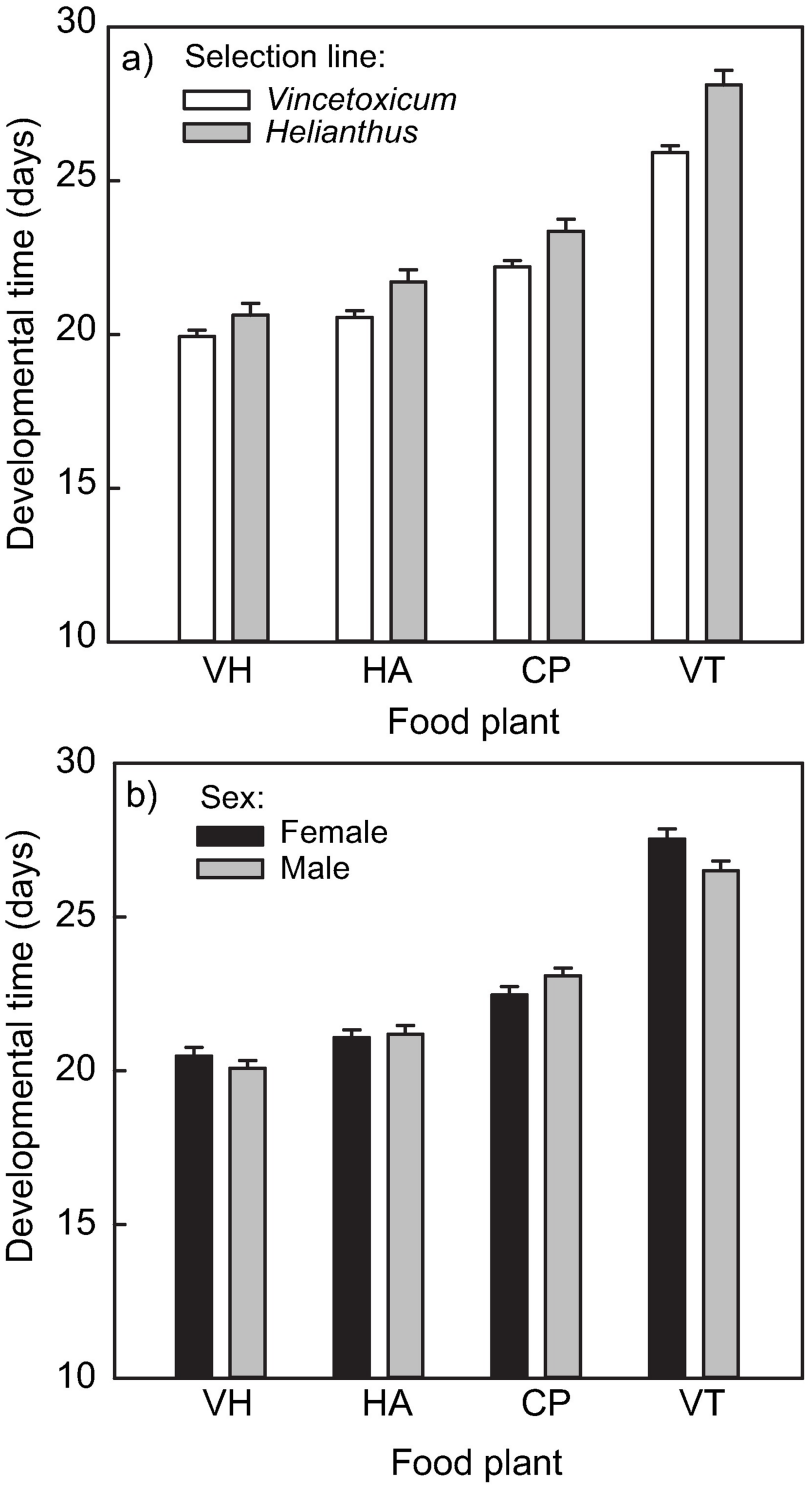
Developmental time of *L*. *equestris* at the end of the selection experiment on different food plants. Interactive effect of a) selection line and food plant, and b) sex and food plant on developmental time of *L*. *equestris*. Food plants are *V*. *hirundinaria* (VH), *H*. *annuus* (HA), *C*. *phrygia* (CP) and *V*. *thapsus* (VT). Values are least square means (± SE).

Overall, the developmental time of *L*. *equestris* was 0.8 days (3.7%) longer in nymphs from the *Helianthus* selection line (Table 3, Fig 5a). However, the interaction between selection line and food plant suggests that the effect of the food-plant species on the developmental time varied between the two selection lines (Table 3 and Fig 5a). Across all food plants, developmental time of *L*. *equestris* from the *Helianthus* selection line was longer compared to the *Vincetoxicum* selection line (Fig 5a), but the difference between the selection lines was most evident on *V*. *thapsus*, on which development was the slowest overall (Fig 5a). Females and males did not differ substantially in developmental time when reared on *V*. *hirundinaria*, *H*. *annuus*, or *C*. *phrygia*, but females feeding on *V*. *thapsus* developed slower than males (food plant × sex; Table 3 and Fig 5b). In addition, developmental times varied among the replicate populations (Table 3). The mean developmental time varied from 21.6 ± 0.17 days to 22.5 ± 0.17 days (least square mean ± SE) among the three replicate populations of *Vincetoxicum* selection line. The mean developmental time was 22.6 ± 0.33 days and 23.3 ± 0.30 days in the two replicate populations (populations 1 and 2) from *Helianthus* selection line.

**Table 3:** Results of ANOVAs testing for the differences in developmental time and adult biomass of *Lygaeus equestris* in a feeding assay.

	Developmental time			Adult biomass		
**Fixed factors**:	df	*F*	*P*	df	*F*	*P*
Selection line	1, 805	12.08	<0.001	1, 807	8.00	0.005
Replicate population (sel. line)	3, 805	2.96	0.031	3, 807	4.98	0.002
Food plant	3, 71	288.55	<0.001	3, 71	574.35	<0.001
Sex	1, 805	1.27	0.260	1, 807	149.25	<0.001
Selection line × food plant	3, 805	3.08	0.027	3, 807	0.57	0.637
Selection line × sex	1, 805	1.06	0.302	1, 807	0.21	0.647
Repl. pop. (sel. line) × food plant	9, 805	1.57	0.119	9, 807	1.41	0.180
Repl. pop. (sel. line) × sex	3, 805	1.06	0.367	3, 807	1.96	0.119
Food plant × sex	3, 805	5.25	0.001	3, 807	16.81	<0.001
Selection line × food plant × sex	3, 805	0.26	0.854	3, 807	1.80	0.146
Repl. pop. (sel. line) × food plant × sex	9, 805	0.61	0.786	9, 807	2.40	0.011
**Random factors**:	df	*X*^*2*^	*P*	df	*X*^*2*^	*P*
Family (repl. pop.)	1	35.24	<0.001	1	12.30	<0.001
Family (repl. pop.) × food plant	1	0.55	0.229	1	0.82	0.182

**Fig 6:**
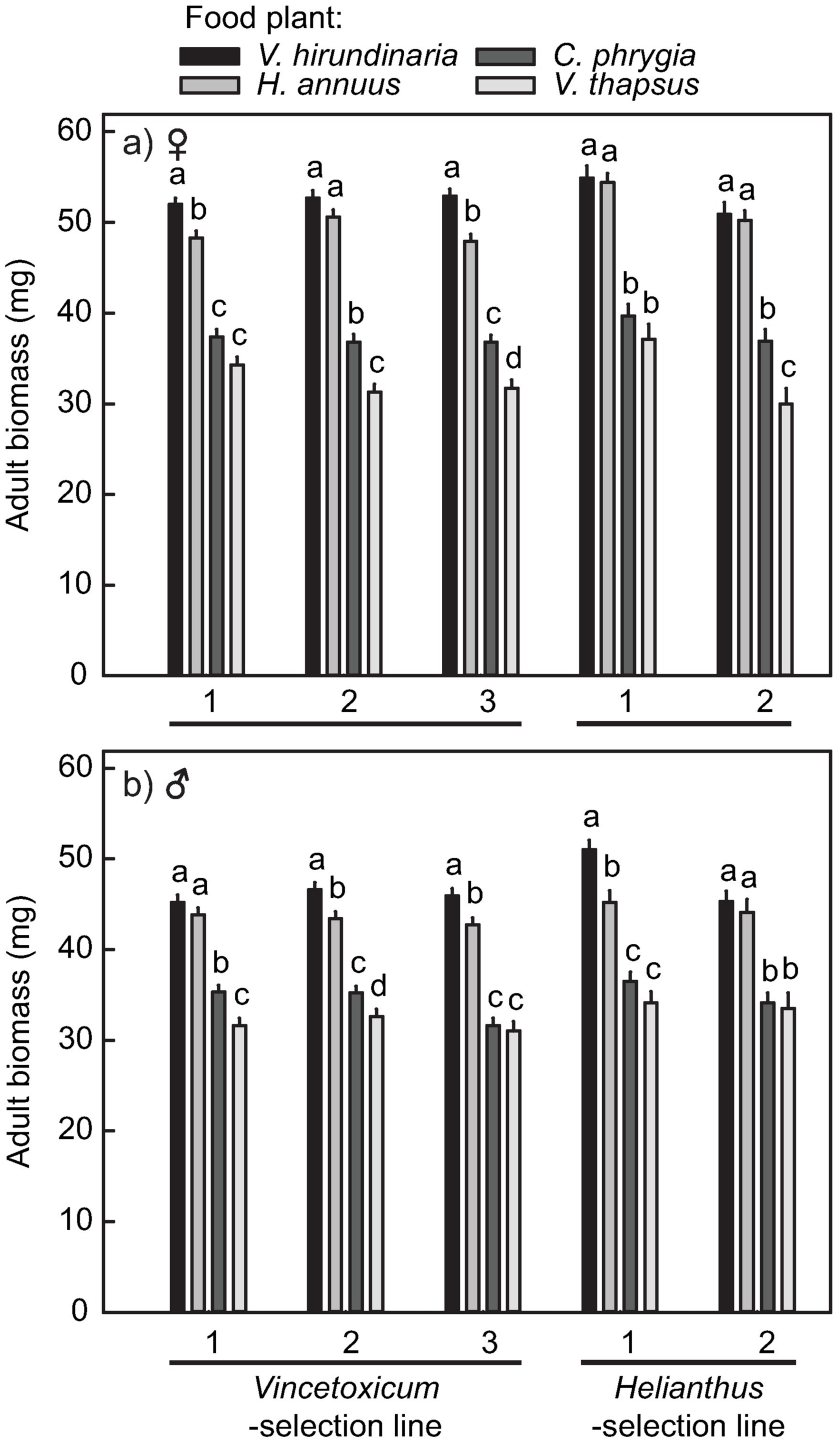
Interactive effect of replicate population, food plant, and sex on adult biomass of *Lygaeus equestris*. The letters indicate significant difference in adult biomass (*P* < 0.05; Tukey’s test) among the food-plant species within each replicate population for a) females and b) males. Values are least square means (± SE).

Adult biomass differed between the selection lines: individuals from *Vincetoxicum* selection line weighted on average 5.4% less than those from the *Helianthus* selection line (Table 3). The three-way interaction between replicate population, food plant, and sex was significant for adult biomass indicating that females and males were differently affected by the food plant, and that these differences between the sexes also varied between the replicate populations (Table 3 and Fig 6). We tested the difference in adult biomass among the food plants within sex and replicate population using Tukey’s test. Adult biomass was higher when the nymphs were fed on *V*. *hirundinaria* and *H*. *annuus* than on the two other food plants. In addition, the effect of these two good-quality food plants on adult biomass varied among the replicate populations (Fig 6). Similarly, we found significant differences in adult biomass between individuals fed on *C*. *phrygia* and *V*. *thapsus* as nymphs only for some of the replicate populations. In general, the differences in adult biomass among individuals fed on the different food plants were greater in females than in males (Fig 6).

We found significant among-family variation in all three fitness traits (mortality χ^2^ = 7.00, df = 1, *P* = 0.004; Table 3). In total, we tested 66 genetic correlations between fitness on different food-plant species but found no statistically significant negative correlations. Instead, we found two significantly positive correlations in the *Vincetoxicum* selection line: developmental time of *L*. *equestris* on *H*. *annuus* correlated positively with developmental time on *V*. *hirundinaria* and *C*. *phrygia* (*r*_s_ = 0.84, n = 21, Bonferroni corrected *P* < 0.006, and *r*_s_ = 0.72, n = 21, *P* = 0.036, respectively). Correlations in the other traits were mostly positive, but not significant.

## Discussion

Selection experiments for host plant specialization, conducted primarily with generalist arthropods, have revealed that host plant specialization can occur over as few as five [6] or 11–12 generations [5,8]. Here we used *L*. *equestris*, a seed-feeding true bug with a clear preference and a strong affiliation with its primary host plant *V*. *hirundinaria* to study whether a more specialized insect can adapt to novel host plants. Our results suggest that despite the ability to occasionally feed on a variety of plant species, *L*. *equestris* is limited in its ability to adapt to novel hosts as we found evidence for adaptation in only one of the four fitness traits for one novel host plant after 20 generations of selection. Adult biomass increased more in bugs reared on *Helianthus* than *Vincetoxicum* over the course of the selection experiment, but we did not observe a similar interaction in the number of fertile eggs, developmental time or mortality leading us to conclude that the signal of adaptation was weak. These results are consistent with the hypothesis of specialization as a dead end because specialization appears to not be easily reverted in this species [48].

In general, directional or stabilizing selection results in loss of genetic variation [5,26] and selection on a novel host plant during our experiment could have lead to loss of quantitative genetic variation in host use. Lack of genetic variation can constrain the evolution of novel host associations [49]. In our previous one-generation split-brood experiment using individuals from the same source population as here, we detected significant quantitative genetic variation in mortality and developmental time of *L*. *equestris* on *V*. *hirundinaria* [25]. In this study, we found that significant quantitative genetic variation still existed after the selection experiment in these fitness traits despite the considerable loss of within population neutral genetic variation, specifically in the *Helianthus* selection line. Quantitative genetic variation is not necessarily linked to neutral genetic variation measured by molecular markers [50] and in general, quantitative traits show reduced heritability only in the smallest and most inbred populations despite loss of neutral genetic variation earlier on [26]. This means that although our experimental populations, especially in the *Helianthus* selection line, lost significant heterozygosity in neutral markers, they had not necessarily lost quantitative genetic variation in the traits that allowed them to use different host plants. Together with results from Laukkanen et al.’s [25] analyses of genetic variation in the same natural population, it appears that quantitative genetic variation was not diminished over the course of selection in our experiment. Thus, the loss of genetic variation as such does not account for the lack of adaptation to the novel host plant *H*. *annuus*.

Even if the loss of genetic variation was not sufficient to lead to the loss of quantitative genetic variation in this study, genetic drift and population bottlenecks may have hindered adaptation to alternative hosts. High genetic differentiation in the *Helianthus* selection line and the negative correlation between pairwise *F*_ST_ and observed heterozygosity suggest that genetic drift had a strong effect on our populations [44,45,51–53] and possibly prevented the fixation of rare beneficial alleles, thereby counteracting selection for novel host use. Because neutral genetic variation was lost only in the selection line for novel host, the loss most likely resulted from the selection regime and not simply from adapting to laboratory conditions. The populations in the *Helianthus* selection line were smaller, and had a smaller effective population size than those in *Vincetoxicum* selection line which could have limited response to selection because selection is less efficient and more variable in small populations [26]. Taken together, it appears that in our study, selection for novel hosts was not strong enough to enable the spread of rare beneficial alleles and counteract the effects of genetic drift. However, the effect of selection was still stronger than that of drift because selection line explained more of the observed variation than the replicate population in our model (Table 3). This suggests that adaptation to novel hosts may occur given sufficient time and large effective population sizes that are not as susceptible to the effects of genetic drift.

Trade-offs theoretically favour the evolution of specialization, as adaptation to one plant species makes populations less adapted to others [3,54], and adaptation to a novel host has sometimes been observed to cause significant reduction in herbivore fitness on an ancestral host [4]. Because we did not find evidence for trade-offs in our earlier one-generation study [25] or in this long-term selection experiment in either of our selection lines, our results suggest that trade-offs do not currently promote host specialization in *L*. *equestris*. A study on host specialization of a seed beetle, *Callosobruchus maculatus*, revealed consistent evidence for trade-offs with a partial genome resequencing approach of the selected lines [55] even though the results from phenotypic assays of trade-offs of the same selection lines were ambiguous [56]. However, unlike in our study, genetic drift did not appear to contribute to the outcome of the selection experiment with *C*. *maculatus* [55] and, therefore, more detailed genomic studies would likely not change the interpretation of our results. Although we found no trade-offs, there was tentative evidence for cost of adaptation as selection for one novel host reduced the ability to feed on yet another alternative, poor quality host plant. When feeding on the poor quality host *V*. *thapsus*, nymphs originating from the *Helianthus* selection line had longer developmental times compared to those from the *Vincetoxicum* selection line.

The fitness of *L*. *equestris* increased on the primary host plant, *V*. *hirundinaria*, in three of the four studied traits during the selection experiment. Because *L*. *equestris* occasionally feeds on alternative host plants when seeds of *V*. *hirundinaria* are scarce, selection to *V*. *hirundinaria* was presumably more extreme during the experiment compared to nature. The increase in fitness may therefore be an indication of trade-offs between the ability to feed on multiple compared to a single host. Alternatively, a part of this increase may be due to adaptation to laboratory conditions that have been shown to select for large size, fast development and increased fecundity in insects [38,57,58].

Specialization of *L*. *equestris* to its primary host *V*. *hirundinaria* appears to be stronger based on this multi-generation study as compared to previous split-brood studies and field observations [23,25]. The ability to use alternative host species may prevent extinction of the populations where the primary host is rare, which would be expected to favour an intermediate level of specialization. Nevertheless, it seems that the exclusive use of alternative host plants is not a feasible long-term strategy, because the nymphs were able to develop on *V*. *thapsus*, but the emerging adults were sterile, and the replicate populations on *C*. *phrygia* survived only for 3–4 generations. Our results suggest that estimates of insect specialization based on single generation studies, especially if fitness is not directly measured, can differ from those based on multigenerational studies.

## Conclusions

In a study by Gould [4] herbivore adaptation to an alternative host plants led to a significant reduction in the fitness on the primary host, but also to adaptation to alternative, taxonomically unrelated, marginal host plants. In contrast to these results, we did not find adaptation to a novel host plant, decrease in fitness on the primary host plant, or trade-offs between different hosts. This suggests that species that are more specialized in host use cannot easily adapt to new host plant species as compared to generalist herbivores. In addition to selection, genetic drift seemed to have caused among-population variation, but these effects were small compared to those of selection for host use. However, random genetic drift likely contributed to the lack of adaptation to novel hosts by inhibiting the spread of beneficial alleles and response to selection. Our results emphasize the need to include species that are somewhat specialized in their host use in studies of the evolution of specialization. They also highlight the power of genetic drift in interfering with population response to selection even in populations that still have ample quantitative genetic variation.

## Supporting information

S1 Appendix Extended methods for establishment of selection lines, microsatellite discovery and genotyping. (PDF)

S1 Table Population sizes and sex ratios of the replicate *Lygaeus equestris* populations. (PDF)

S2 Table Microsatellite marker characteristics. (PDF)

S1 Fig Nymph mortality of *Lygaeus equestris* on four different food plants. (PDF)

S1 Dataset Data for fitness before and after selection for *Vincetoxicum* and *Helianthus* selection lines (number of fertile eggs). (XLSX)

S2 Dataset Data for fitness before and after selection or *Vincetoxicum* and *Helianthus* selection lines (developmental time, adult biomass and mortality). (XLSX)

S3 Dataset Data for genetic variation and cost of adaptation after selection (developmental time, adult biomass and mortality). (XLSX)

S4 Dataset Microsatellite data from the selection experiment. (XLSX)
