# Supplementary material for: Genetic drift precluded adaptation of an insect seed predator to a novel host plant in a long-term selection experiment
Source: PLoS One. 2018 Jun 12;13(6):e0198869. doi: 10.1371/journal.pone.0198869 (PMC5997315; doi:10.1371/journal.pone.0198869)
Supplement: S1 Appendix — (PDF) [file pone.0198869.s001.pdf]

**S1 Appendix. Extended methods for establishment of selection lines, microsatellite discovery and genotyping**

**Establishment of selection lines and replicate populations**

To establish the selection experiment we collected the *Lygaeus equestris* individuals of parental generation from one natural population located in the southwestern archipelago of Finland (N 60°14.0', E 21°56.8') in August 2008. The virgin, new-generation individuals were taken into a laboratory and kept in dark at 4°C to allow them to hibernate. After 10 weeks the temperature was raised to 25°C, and photoperiod adjusted to long-day conditions (22L:2D) to induce mating. The parental generation consisted of 74 females and 66 males, but 25 females and 32 males died before laying eggs. The bugs were allowed to breed randomly in one 12.5 l terrarium. They were fed with a mixture of *Vincetoxicum hirundinaria*, *Helianthus annuus*, *Centaurea phrygia*, and *Verbascum thapsus* seeds. The seeds of *V. hirundinaria* and *V. thapsus* were collected during several growing seasons from several natural populations in the southwestern archipelago of Finland. Seeds from different populations and years were mixed together separately for both plant species. The seeds of *H. annuus* were organic peeled seeds of the brand Urtekram International A/S (Denmark). Organically cultivated *C. phrygia* seeds were obtained from a commercial source (Suomen Niittysiemen Oy and Pratensis Ky, Finland) and soaked for one day in distilled water to soften the husk. Food and distilled water were available *ad libitum*. The parental generation was maintained until the required amount of offspring for the selection experiment was obtained.

After the females had laid eggs, the egg clusters (F1-generation) were transferred from the terrarium to Petri dishes. The nymphs were collected from the Petri dishes in the first day after hatching and randomly divided into a set of populations for the selection experiment. Each egg cluster was first randomly subdivided into four groups to form the four selection lines (*Vincetoxicum*, *Helianthus*, *Centaurea*, and *Verbascum* selection lines). Three replicate populations were established within each selection line to separate the effects of selection from random drift, so that altogether twelve populations were established (see Fig. 1 for experimental set up). To establish the replicate populations for *Vincetoxicum* and *Helianthus* selection lines, 500 first instar nymphs of *L. equestris* of the F1-generation were placed to each of the three terraria within these two selection lines. To establish the three replicate populations for the *Centaurea* and *Verbascum* selection line, 958 nymphs were placed in each terrarium to compensate for the expected greater mortality rate on these food plants. Each replicate population inhabited its own 31 l terrarium that was kept in a climate chamber at 30°C and 22L-2D photoperiod. White garden sand, small rocks, sterilized forest litter, and cotton wool for egg laying were placed on the bottom of each terrarium. The terraria were randomly rotated four times per year within the climate chamber. Two grams of seeds and distilled drinking water were added weekly to each terrarium. Water was available *ad libitum*, but the amount of seeds may have restricted the growth of the replicate populations in *Vincetoxicum* and *Helianthus* selection lines. Dead individuals were removed weekly.

We maintained the *Vincetoxicum* and *Helianthus* selection lines in the laboratory for 26 months, which equals c. 20 generations. At the end of the experiment we estimated the

total number of generations by using generation times specific to each selection line averaged between the beginning and the end of the experiment. Although the average generation time in *Helianthus* selection line was longer by one day, it did not result in fewer generations over the course of the 26 months.

**Microsatellite discovery for *Lygaeus equestris***

Microsatellite discovery and all subsequent genotyping was performed by Center of Evolutionary Applications (University of Turku, Finland). Microsatellites were characterized using a NGS approach where potential microsatellite loci were first located *in silico* from 454 pyrosequencing reads, after which primers were designed and potential loci tested for PCR amplification and polymorphism. Genomic DNA was extracted from muscle tissue with NucleoSpin Tissue kit (Macherey-Nagel). Genomic sequence data was produced by GenoScreen (France) through microsatellite motif enriched shotgun sequencing on Roche's 454 GS-FLX platform (1/32 Titanium plate), using pooled DNA from 11 individuals. As a result 24789 reads were received, out of which 7866 reads had repeat motifs (see below for criteria) and primer design for amplification of microsatellite loci was successful in 434 reads. Primers were designed with software msatcommander 1.0.8 (Faircloth, 2008) for 72 loci containing a di-, tri- or tetranucleotide motif with minimum 8 repeats and amplification was tested using non labelled M13-tailed primers in combination with a dye labelled (FAM, VIC, NED, PET) M13 primer (see e.g. Boutin-Ganache *et al.*, 2001). To improve the microsatellite peak profiles, a GTTT-tail was added to the 5' end of each reverse primer (Brownstein *et al.*, 1996).

A test set of 8 samples was used and each loci was tested in three different PCR conditions with varying annealing temperature. Amplification tests were done in 8 µl reactions containing c. 50-100 ng DNA, 0.1 µM forward primer with M13 tail, 0.2 µM reverse primer, 0.4 µM labelled M13 primer and 1X Qiagen multiplex PCR mastermix. The following two phase PCR profile was used: 95 °C for 15 min, 15 cycles of 94°C for 30 s, annealing at 60, 58 or 56 °C for 1.5 min, 72 °C for 1 min, following with 25 cycles of 94 °C for 30 s, annealing 52 °C for 1.5 min, 72 °C for 1 min and a final extension 72 °C for 15 min. Amplifications were performed on PTC-100 (MJ Research) and AB 2720 (Applied Biosystems) thermal cyclers.

For fragment analysis four PCR products with different dyes were pooled, one microliter from each and diluted with 85 µl of sterile water. 2 µl of the pooled and diluted PCR product was combined with GS600LIZ size standard (Applied Biosystems) and HiDi-formamide (Applied Biosystems). Samples were denatured at 98 °C for three minutes and the size of the fragments was determined by capillary electrophoresis on an ABI Prism$^{TM}$ 3130*xl* genetic analysis instrument. Peak profiles were visually checked using GeneMapper version 4.0 (Applied Biosystems) and each locus was categorized as having "good amplification, non-specific amplification and weak/no amplification". Depending on assigned category, the annealing temperature for each locus was either increased or decreased for the second and similarly again for third test round.

A larger preliminary data set (N = 48) was created with all loci that could be confidently genotyped and had two or more alleles. Based on this dataset we chose 18 markers that could be combined in one panel, i.e. analyzed simultaneously in one Abi

fragment analysis, and reordered the forward primers with labels to replace the M13-tailed primers used earlier. More testing was done to optimize a multiplexed PCR panel, including several PCR tests with minor modifications to equalize peak intensities, minimize non-specific amplification and confirm repeatability. Two markers were left out due to difficulties in allele determination. The final microsatellite panel consisted of 16 markers and could be amplified in three multiplexed reactions and one singleplex reaction. We used Micro-Checker 2.2.3 (Van Oosterhout et al. 2004) to test for the presence and frequency of null alleles with the method of Brookfield 1. The results indicated presence of null alleles in four loci that were then excluded from further analysis.

**Microsatellite genotyping**

DNA was extracted from whole individuals stored in 70 % ethanol and kept in + 4 °C. The head and thorax were dissected and used for DNA extraction with a silica-based purification method modified from Elphinstone *et al.*, (2003). DNA was diluted 1:7 with sterile water and samples were genotyped with 12 microsatellite markers (Lyga-04, Lyga-05, Lyga-13, Lyga-16, Lyga-18, Lyga-32, Lyga-44, Lyga-52, Lyga-53, Lyga-55, Lyga-61, Lyga-62; Table S2). To improve the microsatellite peak profiles, a GTTT-tail was added to the 5' end of each nonlabelled primer (Brownstein *et al.*, 1996).

PCR amplification was carried out in three 8 µl multiplexed reactions (MP1-3) and one singleplex (S) reaction using QIAGEN Multiplex PCR Kit (Qiagen Inc. Valencia, CA, USA) with the annealing temperature of 55, 58 and 60 °C. Primer concentrations varied from 0.08 to 0.3 µM. PCR profile was according to the manufacturer's standard protocol for microsatellites. Amplifications were performed on PTC-100 (MJ Research) and AB 2720 (Applied Biosystems) thermal cyclers. For electrophoresis the PCR products were pooled by combining 0.8 to 1.0 µl of each multi- or singleplexed PCR and diluted with 100 µl of sterile water. 2 µl of the pooled and diluted PCR product was combined with GS600LIZ size standard (Applied Biosystems) and HiDi-formamide (Applied Biosystems). Samples were denatured at 98 °C for three minutes and the size of the fragments was determined by capillary electrophoresis on an ABI PrismTM 3130xl genetic analysis instrument.

The genotypes were scored using GeneMapper version 4.0 (Applied Biosystems) and following visual inspection, exported to a spreadsheet program for downstream analyses.
Genotyping error was assessed by repeating the PCR amplification of 16 individuals, genotyping these samples separately and then comparing to the original genotypes. Direct count genotyping error rate per allele per locus varied 0 – 3.3 %, mean overall loci 0.2 %.

**References**

Boutin-Ganache, I., Raposo, M., Raymond, M. & Deschepper, C.F. 2001. M13-tailed primers improve the readability and usability of microsatellite analyses performed with two different allele-sizing methods. *BioTechniques* **31**: 24–28.

Brownstein, M.J., Carpten, J.D. & Smith, J.R. 1996. Modulation of non-templated nucleotide addition by Taq DNA polymerase: primer modifications that facilitate genotyping. *BioTechniques* **20**: 1004–10010.

Elphinstone, M.S., Hinten, G.N., Anderson, M.J. & Nock, C.J. 2003. An inexpensive and high-throughput procedure to extract and purify total genomic DNA for population studies. *Mol. Ecol. Notes* **3**: 317–320.

Faircloth, BC 2008. MSATCOMMANDER: detection of microsatellite repeat arrays and automated, locus-specific primer design. *Mol. Ecol. Resour.* **8**: 92–94.

Van Oosterhout C, Hutchinson WF, Wills DPM, Shipley P. (2004) MICRO-CHECKER: Software for identifying and correcting genotyping errors in microsatellite data. *Mol. Ecol. Notes* **4**: 535–538.
