# Supplementary material for: Genetic drift precluded adaptation of an insect seed predator to a novel host plant in a long-term selection experiment
Source: PLoS One. 2018 Jun 12;13(6):e0198869. doi: 10.1371/journal.pone.0198869 (PMC5997315; doi:10.1371/journal.pone.0198869)
Supplement: S1 Table — (PDF) [file pone.0198869.s002.pdf]

S1 Table. Population sizes and sex ratios of the replicate *Lygaeus equestris* populations

Population sizes, sex ratios, and effective population sizes were recorded after 22 months of selection (c. 17 generations). Calculations assumed non-overlapping generations.

Selection line	Replicate population	Population size	Females (%)	Effective population size
V. hirundinaria	1	441	43	432
	2	257	33	227
	3	327	65	298
H. annuus	1	277	63	258
	2	307	49	307
	3	62	46	62
