# Supplementary material for: Genetic drift precluded adaptation of an insect seed predator to a novel host plant in a long-term selection experiment
Source: PLoS One. 2018 Jun 12;13(6):e0198869. doi: 10.1371/journal.pone.0198869 (PMC5997315; doi:10.1371/journal.pone.0198869)
Supplement: S2 Table — (PDF) [file pone.0198869.s003.pdf]

S2 Table. Microsatellite marker characteristics

Characteristics of 12 newly developed polymorphic microsatellite loci for Lygaeus equestris. GenBank accession number, forward and reverse primer sequence (*), repeat motif, range of allele sizes (bp), numberr of alleles, PCR multiplex group, annealing temperature (Ta), fluorescent label used and primer concentration are given for each locus. A total of 30 individuals from Lammasluoto (N 60°14.0', E 21°56.8') population were screened.

Locus	GenBank Accession nr	Sequence 5' - 3'	Repeat motif	Allele range (bp)	Nr of alleles	PCR multiplex group	Ta	Fluorescent label	Primer concentration (µM) in reaction
Lyga-04	MH211603	F: CGTTTCTGAGTTACTGCGACC<br>R: **GTTT**AAAGCATGGTCCCACAAAGTG	AC	87 - 107	8	mp1	60	ned	0.1
Lyga-05	MH211604	F: TGAAGAGGCCAAGGTCCATG<br>R: **GTTT**GAACCTGTTAGCCACACAG	AAG	101 - 104	2	mp2	58	vic	0.08
Lyga-13	MH211605	F: GCTGCAACCTTCTCGTATATTT<br>R: **GTT**GGCATATGCTACTGAGAATACCC	AC	172 - 203	8	single	58	pet	0.2
Lyga-16	MH211606	F: GACACCCAATTAGTCGCATGG<br>R: **GTTT**AACTGGCCTGTAGAGCGTTG	AC	156 - 180	7	mp1	60	vic	0.2
Lyga-18	MH211607	F: TCGGACTGTAACGTAGCCTG<br>R:**GTTT**ATTTCATCCAATCGAGATCTGAGG	AGAT	159 - 187	3	mp2	58	fam	0.1
Lyga-32	MH211608	F: CCACAGCTCTAAATTCACTCCAC<br>R: **GTTT**CCTCGACGCTAGAAATGCC	AAG	305 - 311	3	mp2	58	fam	0.2
Lyga-44	MH211609	F: GCTGATTAAGACTGGGAAGGC<br>R: **GTTT**CTTACAACCTCATTCCTGCC	AAT	322 - 328	4	mp3	55	vic	0.15
Lyga-52	MH211610	F: CCTCTTCTCACATATCCTCAGTC<br>R: **GTTT**CCCTGATCCAGGGCAAAT	TC	92 - 94	2	mp1	60	pet	0.08
Lyga-53	MH211611	F: GTCATGAATTGTGTTTCATAAAGATT<br>R: **GTTT**CAGGAGGCCCTAATCAACA	CA	111 - 121	5	mp1	60	pet	0.2
Lyga-55	MH211612	F: CCTTAATATCAAGTGCCCGC<br>R: **GTTT**GAGGTCCACCGAGTTCAT	CA	79 - 104	5	mp1	60	fam	0.2
Lyga-61	MH211613	F: AAATCTTGAGTTTTACCCCGTT<br>R: **GTTT**GCACAAAACCTTAAATAACGCC	GT	246 - 257	3	mp2	58	fam	0.2
Lyga-62	MH211614	F: ATTGTTACACTTGGGACGGA<br>R: **GTTT**CAGGTTGACCTTTCGGGTA	AC	118 - 127	3	mp1	60	fam	0.3

* reverse primers have added nucleotides (GTTT) in the 5’ end.
