# Supplementary material for: Genetic drift precluded adaptation of an insect seed predator to a novel host plant in a long-term selection experiment
Source: PLoS One. 2018 Jun 12;13(6):e0198869. doi: 10.1371/journal.pone.0198869 (PMC5997315; doi:10.1371/journal.pone.0198869)
Supplement: S1 Fig — (PDF) [file pone.0198869.s004.pdf]

S1 Figure. Nymph mortality of *Lygaeus equestris* on four different food plants

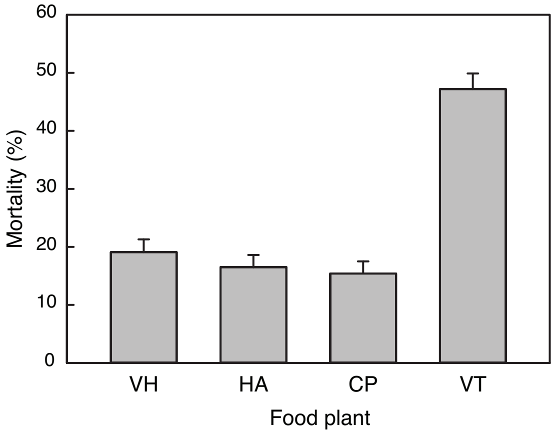

Nymph mortality of *L. equestris* on the primary food plant *V. hirundinaria* (VH), and three novel food plants *H. annuus* (HA), *C. phrygia* (CP) and *V. thapsus* (VT) at the end of the selection experiment.
